# The Association between Prenatal Psychosocial Stress and Blood Pressure in the Child at Age 5–7 Years

**DOI:** 10.1371/journal.pone.0043548

**Published:** 2012-08-21

**Authors:** Aimée E. van Dijk, Manon van Eijsden, Karien Stronks, Reinoud J. B. J. Gemke, Tanja G. M. Vrijkotte

**Affiliations:** 1 Department of Public Health, Academic Medical Center-University of Amsterdam, Amsterdam, The Netherlands; 2 Department of Epidemiology, Documentation and Health Promotion, Public Health Service of Amsterdam (GGD), Amsterdam, The Netherlands; 3 Institute of Health Sciences, VU University, Amsterdam, The Netherlands; 4 Department of Pediatrics, VU University Medical Center, Amsterdam, The Netherlands; Erasmus Medical Center, The Netherlands

## Abstract

**Objective:**

Prenatal maternal stress could have permanent effects on the offspring’s tissue structure and function, which may predispose to cardiovascular diseases. We investigated whether maternal psychosocial stress is a prenatal factor affecting the blood pressure (BP) of offspring.

**Study Design:**

In the Amsterdam Born Children and their Development (ABCD) study, around gestational week 16, depressive symptoms, state-anxiety, pregnancy-related anxiety, parenting daily hassles and job strain were recorded by questionnaire. A cumulative stress score was also calculated (based on 80^th^ percentiles). Systolic and diastolic BP and mean arterial pressure (MAP) were measured in the offspring at age 5–7 years. Inclusion criteria were: no use of antihypertensive medication during pregnancy; singleton birth; no reported cardiovascular problems in the child (N = 2968 included).

**Results:**

After adjustment for confounders, the single stress scales were not associated with systolic and diastolic BP, MAP and hypertension (p>0.05). The presence of 3–4 psychosocial stressors prenatally (4%) was associated with 1.5 mmHg higher systolic and diastolic BP (p = 0.046; p = 0.04) and 1.5 mmHg higher MAP in the offspring (p = 0.02) compared to no stressors (46%). The presence of 3–4 stressors did not significantly increase the risk for hypertension (OR 1.8; 95% CI 0.93.4). Associations did not differ between sexes. Bonferroni correction for multiple testing rendered all associations non-significant.

**Conclusions:**

The presence of multiple psychosocial stressors during pregnancy was associated with higher systolic and diastolic BP and MAP in the child at age 5–7. Further investigation of maternal prenatal stress may be valuable for later life cardiovascular health.

## Introduction

According to the ‘fetal programming hypothesis’, prenatal exposure to suboptimal intra-uterine conditions could predispose the individual to chronic disease at adult age [1]. Such conditions can be maternal stress or the administration of stress hormone (glucocorticoid), which alters fetal growth and has permanent effects on the tissue structure and function of the offspring [2]. For example, the stress response and the renin-angiotensin system may be affected which can lead to adult risk of cardiovascular disease [3]–[18].

Several mechanisms underlying the association between stress conditions such as psychosocial stress during pregnancy and fetal programming have been proposed. One involves inflammation markers: maternal psychosocial factors can contribute to increased inflammation during pregnancy [19], which influences the development of adult cardiovascular disease, although this mechanism has not been extensively investigated [20]. Another potential pathway requires the influence of psychosocial stress on the maternal hypothalamic-pituitary-adrenal (HPA) axis: This leads to hypersecretion of the glucocorticoid cortisol, which may influence the development of the fetal HPA axis [6], [7], and immune function [21] as it partly crosses the placenta. Alternatively, or additionally, maternal anxiety may increase the permeability of the placenta to cortisol due to downregulation of 11-Beta hydroxysteroid dehydrogenase [22]–[24]. Hyperactivity of the offspring’s HPA axis is in turn associated with risk factors for cardiovascular diseases such as increased blood pressure (BP), insulin resistance, glucose intolerance and hyperlipidemia [25].

From studies in rodents and sheep we also know that prenatal glucocorticoid exposure is associated with reductions in nephron numbers [17], [18], changes in vascular responsivity to vasoconstrictors [12], locally enhanced activity of the renin-angiotensin system in the kidney and brain, and alterations of the baroreceptor response [5], [13]; factors that all contribute to the onset of hypertension. Convincing evidence comes both from animal studies, in which prenatal exposure to excess glucocorticoids results in persistent elevation of arterial BP in adulthood, with potential sex differences (for review, [26]), and from human studies. For example, antenatal glucocorticoid administration is linked with high BP in the fetus [27] and in adolescence [28]. Those studies have, however, been complicated by high doses of glucocorticoids, which are not comparable to levels normally occurring during pregnancy. In addition, glucocorticoid treatment usually takes place when pre-term birth is expected, adding bias because of the complications of premature birth.

To our knowledge, there are no previous human studies investigating a potential association between naturally occurring psychosocial stress and offspring blood pressure. Therefore, our aim was to study maternal psychosocial stress as a potential disruptor of BP in offspring. Psychosocial complaints are important to investigate because they are highly prevalent in what are otherwise normal pregnancies [29]. Psychosocial stress is accompanied by increases in stress hormones that occur naturally [7], [16], [30] with the potential to program the fetus. Tse et al. [31] reported an association between cumulative stress during pregnancy and cortisol releasing hormone (CRH) concentrations, in separate analyses of Blacks and Hispanics. These findings indicate that the cumulation of stress heightens allostatic load, as posed by McEwen and Stellar in 1993 [32].

The present study was designed to, for the first time, examine the relation between maternal prenatal psychosocial stress and BP in the child at age five-seven, and possible effect-modification by sex, given the accumulating evidence of sex-specific effects in fetal programming [33]–[37]. We hypothesized that the presence of psychosocial stressors is associated with higher systolic and diastolic BP, mean arterial pressure (MAP) and a higher prevalence of hypertension in the offspring at age 5–7. The results of this study may open up new avenues for preventive strategies with regard to hypertension.

## Methods

The present study is part of the Amsterdam Born Children and their Development (ABCD) study, a prospective, longitudinal birth cohort [38]. The main goal of the ABCD study is to examine and determine factors in early life (during pregnancy and infancy) that might explain the later health of the child, and the differences in health between children (www.abcd-study.nl). Approval was obtained from the medical ethical committees of all participating hospitals and the Registration Committee of Amsterdam. All participants gave written informed consent for themselves and their children.

### Study Population

**Figure 1:**
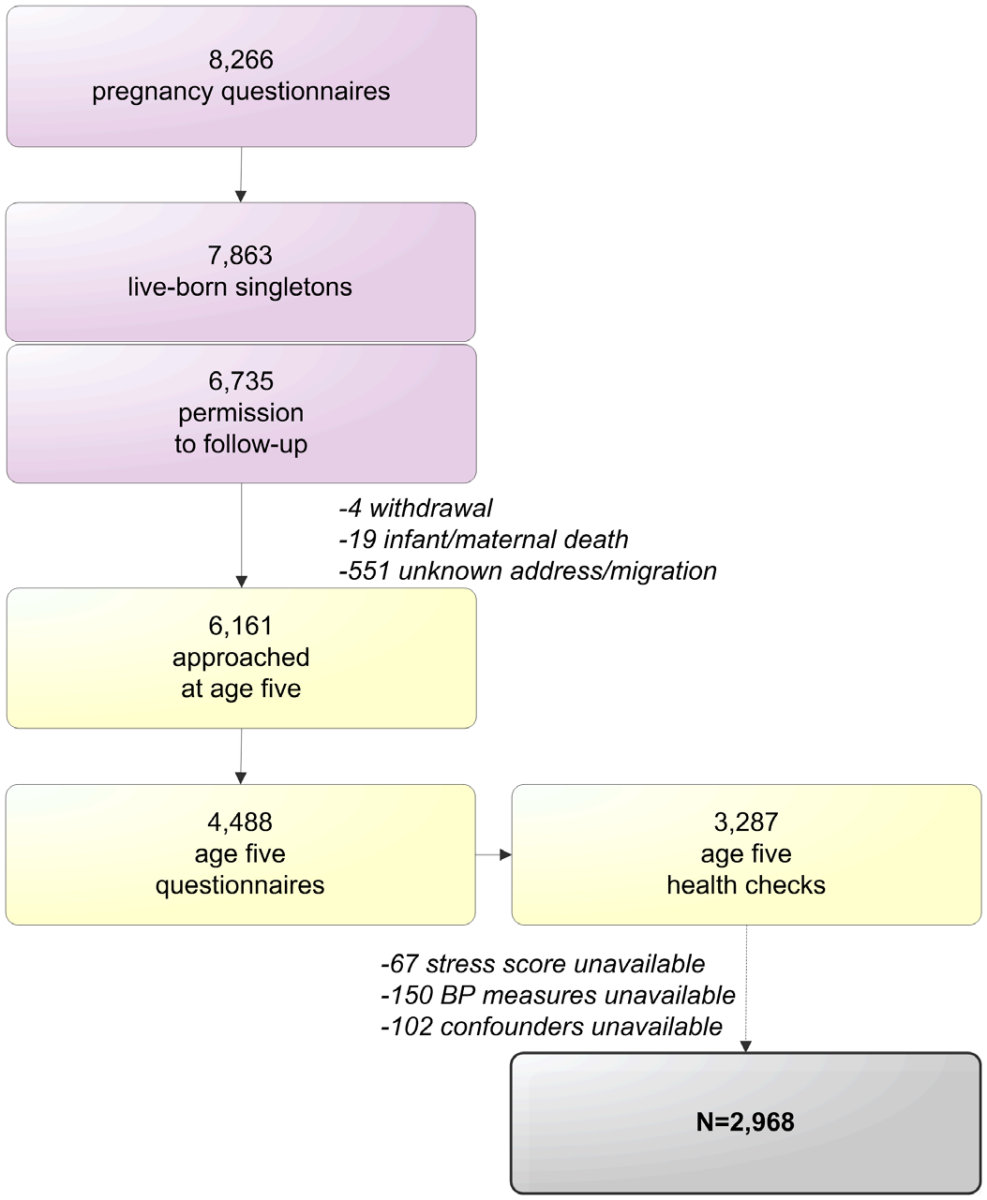
Procedure of the ABCD Study cohort and inclusion in the current analyses.

The selection of the current study population is visualized in Figure 1. In 2003–2004, 12 373 Amsterdam women who first attended antenatal care in Amsterdam were approached to participate. Of these women, 8266 (67%) returned the pregnancy questionnaire, which included multiple psychosocial stress instruments (phase 1). For singleton live births, 6735 mothers gave permission for follow-up. When the children turned five, the addresses of 6161 mothers were retrieved from the Youth Health Care registry; attrition in this follow-up number was largely due to untraceable changes in address or migration. The mothers received a questionnaire, including an informed consent sheet for a health check of their child, which 4488 women returned. The health check itself consisted of various health measurements in 3287 children (2008–2010; mean age 5.7) [39].

The current study population included only mother-child pairs of which the mother had filled out the psychosocial questions (using validated instruments) during pregnancy and the child participated in the age five health check, specifically for the BP measurements. Additionally, data on all covariates had to be available. None of the included children had Down’s syndrome or cardiovascular conditions (reported in the age five questionnaire). None of the included mothers reported the use of antihypertensive medication. Following these criteria, 2968 mother-child pairs were included in the current study’s analysis (37.7% follow-up of live-born singletons).

### Independent Variables: Maternal Prenatal Psychosocial Stress

The included mothers filled out the pregnancy questionnaire around mean gestational week 16 (median 16 standard deviation 4 interquartile range 13–19 minimum 6 maximum 40).

#### State anxiety

Anxiety was assessed using the Dutch version [40] of the State-Trait Anxiety Inventory (STAI) [41]. The 20 items regarding state anxiety (transient or temporarily experienced anxiety over the preceding week) were included in our questionnaire, with each item scored on a 4-point scale (0 = rarely or none of the time, 1 = some or a little of the time, 2 = occasionally or a moderate amount of the time and 3 = most or all of the time). In the present sample the internal consistency (Cronbach’s alpha) of the STAI scale was 0.94.

#### Depressive symptoms

Depressive symptoms were assessed using the validated Dutch version of the 20-item Center for Epidemiological Studies Depression Scale (CES-D) [42], [43]. This scale evaluates the frequency of depressive symptoms experienced over the preceding week. Each item was scored on a four-point scale (0 = rarely or none of the time, 1 = some or a little of the time, 2 = occasionally or a moderate amount of the time and 3 = most or all of the time). Internal consistency (Cronbach’s alpha) of the scale was 0.90.

#### Pregnancy-related anxiety

Pregnancy anxiety was assessed using an abbreviated 10-item version [44] of the Pregnancy Related Anxieties Questionnaire (PRAQ) [45]. Each item was scored on a four-point scale (0 = definitely not true, 1 = not true, 2 = true and 3 = very true). Three aspects that can be distinguished are ‘fear of giving birth’, ‘fear of bearing a physically or mentally handicapped child’ and ‘concern about one’s appearance’, but in the current study only the overall score was utilized. Internal consistency (Cronbach’s alpha) of the scale was 0.79.

#### Parenting stress

To assess parenting stress a Dutch adaptation [46] of the 20-item Parenting Daily Hassles (PDH) scale was used [47]. The parents rated the occurrence of typical everyday events in parenting and parent-child interactions on a four-point scale (0 = never or rarely, 1 = sometimes, 2 = a lot and 3 = constantly). Women with no previous children scored zero on this scale by default. Internal consistency (Cronbach’s alpha) of the scale was 0.84.

#### Job strain

To assess job strain (or work stress), a Dutch version of the Job Content Questionnaire was used [48], [49]. It consists of 2 subscales: job demands and job control. The job demands subscale consists of 25 four-point scale items focusing on work pace, mental workload and physical workload. The job control subscale consists of 11 items. In concordance with the JCQ-guidelines, we divided the job demand score into: low (<50th percentile), moderate (50th-80th percentile) and high (>80th percentile). Similarly, we divided the job control score into high (>50th percentile), moderate (20th–50th percentile), and low (<20th percentile). Women in the category ‘low job strain’ had reported low job demand with moderate or high job control. ‘High job strain’, consisted of women who reported high job demand with low or moderate job control. All other women fell into the ‘moderate job strain’ category. Internal consistency (Cronbach’s alpha) of the workload scale was 0.85, and internal consistency of the work control scale was 0.93.

#### Cumulative stress score

A total, cumulative stress score was calculated by ascribing points to the number of times a mother ends up above the 80th percentile of three of the above mentioned stress scales (depressive symptoms, pregnancy-related anxiety and parenting stress). A fourth point is added if the mother also scored high on the job strain scale. This resulted in a sum score between 0 and 4, which was divided into three categories: No stress (0 stressors), 1 stressor, 2 stressors, and 3–4 stressors (3 and 4 taken together because of low numbers). State anxiety was not included in the total score because of its high correlation with depressive symptoms (correlation coefficient 0.9).

### Dependent Variable: Blood Pressure in the Child

BP was measured with the automatic oscillometric method, using the Omron 705 IT (Omron Healthcare Inc, Bannockburn, IL, USA) with a small cuff (arm circumference 17–22 cm) on the non-dominant arm. The BP measurement was part of a larger procedure, which has been described previously [39]. The child was seated at a table, for five minutes, after which BP was measured twice. When either the systolic or diastolic pressure differed more than 10 mmHg between the two measurements, a third measurement was taken (n = 502; 17%). In the current study, the average of the two measurements closest together was used. MAP was calculated using the following equation: MAP = Diastolic pressure +1/3 (systolic pressure-diastolic pressure).

Hypertension was defined using guidelines from the Fourth Report on the Diagnosis, Evaluation, and Treatment of High Blood Pressure in Children and Adolescents (40). Following those guidelines, first the sex-specific and age-specific height (based on age five assessment) Z-scores were calculated within our own population (a Z-score, or standard score, indicates by how many standard deviations an observation is above or below the mean), which we then used in the regression equations provided in the report. The regression models calculated each child’s BP Z-score (and the matching BP percentile) that could then be dichotomized into normotension (≤p95 on systolic and diastolic BP) and hypertension (>95^th^ percentile on systolic or diastolic BP).

### Covariates

The origins and definitions of most of the covariates have been described in more detail previously [39]. Maternal age, ethnicity, pre-pregnancy weight and height (BMI, kg/m^2^), educational level (years of education after primary school; proxy for socioeconomic status), smoking, alcohol consumption and pre-existing conditions (dichotomous: none vs. one or more, including hypertension, diabetes, hypothyroidism, hyperthyroidism and epilepsy) in pregnancy were available from the pregnancy questionnaire, hence all self-reported. Ethnicity was defined by maternal country of birth (in line with the definition by Statistics Netherlands (CBS)) and categorized into 8 categories (Dutch; Surinamese; Antillean; Turkish; Moroccan; Ghanaian; Other non-western country; Other western country). Smoking during pregnancy was categorized into non-smoking, 1–5 cigarettes/day and > = 6 cigarettes/day. Alcohol consumption during pregnancy was dichotomized (yes/no). Pregnancy-induced hypertension (dichotomous: yes/no) was available by combining data from the questionnaire and Dutch Perinatal Registration (PRN, www.perinatreg.nl) and classified in accordance with the guidelines of the International Society for the Study of Hypertension in Pregnancy (www.isshp.com). Parity (primiparous: yes/no), gestational age at birth, birth weight and sex were available from the PRN and Youth Health Care Registration. Gestational age was based on ultrasound by the obstetric care provider or, when unavailable (<10%), on the first day of the last menstrual period. The height and weight of the child were measured at the age five health check, from which BMI was calculated. Maternal (family) hypertension was also included and was defined as being present when the mother either answered ‘yes’ to the question ‘Have you ever had high blood pressure?’ or ‘Does or did anyone in your immediate family (parents, brothers and sisters) have high blood pressure before the age of 55?’ in the questionnaire at age five. Paternal (family) hypertension was defined as being present when the mother either answered ‘yes’ to the question ‘Has your child’s biological father ever had high blood pressure?’ or ‘Does or did anyone in the child’s biological father’s immediate family have high blood pressure before the age of 55?’.

### Statistics

Associations between stress scales and blood pressure were explored using both linear (systolic BP, diastolic BP and MAP) and logistic (hypertension) regression models (SPSS 18.0, SPSS Inc., Chicago, USA). Regression analyses were adjusted for sex, height and age of the child by default (model 1). Analyses regarding hypertension were exempt from this rule, because hypertension was already defined by sex, age and height-specific rules. All potential confounders were determined a priori and added simultaneously (model 2): maternal and paternal (family) hypertension, maternal age, ethnicity, pre-pregnancy BMI, educational level, primiparity, pre-existing conditions, pregnancy-induced hypertension, smoking, alcohol consumption, gestational age, birth weight and BMI child (as in [50]). Effect-modification by sex was tested by adding an interaction term (stress measure * sex) to model 2. Associations were also checked for linearity using restricted cubic splines in R (R Foundation for Statistical Computing), and all associations met the assumption of linearity.

Additionally, we checked whether gestational age at pregnancy (stress) assessment could be a confounder in the analyses. ANOVA and Spearman’s correlation coefficients showed no significant association between gestational time point and the independent variables (maternal stress), and no association between gestational time point and the dependent variables (offspring blood pressure).

## Results

The characteristics of the mothers, fathers and children included in this study are presented in Table 1. The women in the present study had a mean age of 31.9 years, mean BMI of 22.9 kg/m^2^ and 76.2% was of Dutch origin. High job strain was experienced by 14.2%, and 4.2% experienced 3 or 4 psychosocial stressors. Blood pressure of the children was measured at a mean age of 5.7 years. They had a mean BMI of 15.5 kg/m^2^, mean systolic BP of 97.7 mmHg and mean diastolic BP of 58.0 mmHg. Compared to the women from the initial total sample not included in the current study, the sample included in the current study (N = 2968) had significantly lower mean levels of depressive symptoms (Δ0.5), anxiety (Δ0.8), higher pregnancy-related anxiety (Δ–0.7) and lower parenting daily hassles (Δ1.1) (all p<0.01).

**Table 1:** Maternal/paternal and child characteristics (N = 2968).

	Mean/Percentage	SD	Interquartile range	
Maternal/paternal			Lower	Upper
Age (y)	31.9	4.6	26.9	36.9
*Ethnicity*				
Dutch (%yes)	76.2			
Surinamese (%yes)	3.7			
Antillean (%yes)	0.7			
Turkish (%yes)	2.3			
Moroccan (%yes)	4.0			
Ghanaian (%yes)	1.0			
Other non-western country (%yes)	5.5			
Other western country (%yes)	6.6			
Pre-pregnancy BMI (kg/m^2^)	22.9	3.8	18.9	26.9
Obesity (BMI≥30) (%yes)	5.0			
Education after primary school (y)	9.8	3.6	4.8	14.8
Primiparous (%yes)	55.8			
Pregnancy hypertension (%yes)	8.9			
Pre-existing condition (%yes)	4.5			
*Smoking:*				
Non-smoking (%yes)	90.9			
1–5 cigarettes/day (%yes)	6.2			
> = 6 cigarettes/day (%yes)	2.9			
Alcohol (%yes)	27.3			
Maternal (family) hypertension (%yes)	22.9			
Paternal (family) hypertension (%yes)	17.7			
**Maternal-Stress**				
Depressive symptoms	12	8	2	22
State anxiety	37	10	24	50
Pregnancy-related anxiety (total score)	20	5	15	25
Parenting daily hassles*	36	7	26	46
*Job strain:*				
No job (%yes)	24.8			
Low job strain (%yes)	19.0			
Moderate job strain (%yes)	42.0			
High job strain (%yes)	14.2			
*Cumulative stress score:*				
No stress (%yes)	46.0			
1 Stressor (%yes)	35.0			
2 Stressors (%yes)	14.8			
3–4 Stressors (%yes)	4.2			
**Child – At birth**				
Sex (%boys)	50.2			
Gestational age (weeks)	39.9	1.7	38.0	41.8
Birthweight (g)	3478	549	2805	4151
**Child – At age 5 measurement**				
Age (y)	5.7	0.5	5.0	6.5
Height (cm)	117	6	109	124
BMI (kg/m^2^)	15.5	1.5	13.8	17.2
Systolic blood pressure (mmHg)	97.7	8.6	87.2	108.2
Diastolic blood pressure (mmHg)	58.0	7.9	49.0	67.0
Mean Arterial Pressure (mmHg)	71.3	7.4	62.8	79.8
Hypertension (%yes)	6.1			

* Parenting daily hassles were only analyzed on the subgroup of mothers who were already raising one or more children (43.9%; n = 1303).

### Single Stress Scales

Depressive symptoms were associated with higher systolic BP, diastolic BP and MAP in the first, minimally adjusted regression models (p = 0.045; p<0.01 and p<0.01 respectively) (Table 2). These associations were attenuated and lost significance upon full adjustment for confounders (model 2).

**Table 2:** Associations (β coefficients) between maternal prenatal stressors and blood pressure measures Systolic Blood Pressure, Diastolic Blood Pressure and Mean Arterial Pressure in the child at age five-seven (N = 2968).

	Model 1*		Model 2†		Model 1		Model 2		Model 1		Model 2	
	β	95%CI	β	95%CI	β	95%CI	β	95%CI	β	95%CI	β	95%CI
	Systolic BP				Diastolic BP				MAP			
Depressive symptoms^a^	0.04**	(0.00;0.07)	0.01	(−0.03;0.05)	0.06**	(0.02;0.09)	0.01	(−0.02;0.05)	0.05**	(0.02;0.08)	0.01	(−0.02;0.05)
State anxiety^a^	0.05**	(0.02;0.08)	0.03	(−0.01;0.06)	0.06**	(0.03;0.09)	0.03	(0.00;0.05)	0.05**	(0.03;0.08)	0.03	(0.00;0.05)
Pregnancy-related anxiety^a^	0.09**	(0.03;0.15)	0.03	(−0.04;0.10)	0.06	(0.00;0.12)	−0.01	(−0.07;0.05)	0.07**	(0.01;0.12)	0.00	(−0.05;0.06)
Parenting daily hassles Ħ^a^	0.03	(−0.03;0.09)	0.02	(−0.04;0.08)	0.04	(−0.01;0.09)	0.02	(−0.03;0.08)	0.04	(−0.02;0.09)	0.02	(−0.03;0.07)
*Job strain^b^*												
Low job strain (reference)	0		0		0		0		0		0	
No job	0.5	(−0.4;1.4)	−0.1	(−1.0;0.8)	1.2**	(0.4;2.0)	0.2	(−0.7;1.1)	1.0**	(0.2;1.8)	0.1	(−0.7;0.9)
Moderate job strain	0.0	(−0.8;0.8)	0.1	(−0.7;0.9)	0.1	(−0.7;0.9)	0.1	(−0.7;0.8)	0.1	(−0.6;0.8)	0.1	(−0.6;0.8)
High job strain	0.0	(−1.1;1.0)	−0.3	(−1.3;0.7)	0.6	(−0.4;1.5)	0.1	(−0.8;1.1)	0.4	(−0.5;1.3)	0.0	(−0.9;0.9)
*Cumulative stress score^c^*												
No stress (reference)	0		0		0		0		0		0	
1 Stressor	0.2	(−0.5;0.8)	0.1	(−0.5;0.8)	0.4	(−0.2;1.0)	0.2	(−0.4;0.8)	0.3	(−0.3;0.9)	0.2	(−0.4;0.8)
2 Stressors	−0.4	(−1.2;0.5)	−0.7	(−1.6;0.2)	0.1	(−0.7;0.9)	−0.6	(−1.4;0.3)	−0.1	(−0.8;0.7)	−0.6	(−1.4;0.2)
3–4 Stressors	2.2**	(0.7;3.7)	1.5**	(0.0;3.0)	2.5**	(1.1;3.9)	1.5**	(0.1;2.9)	2.4**	(1.1;3.7)	1.5**	(0.2;2.8)

** p<0.05.

* To model 1, sex, height and age of the child at measurement were added as covariates.

† To model 2, additionally added covariates are: maternal and paternal (family) hypertension, maternal age, ethnicity, pre-pregnancy BMI, educational level, primiparity, pre-existing conditions, pregnancy-induced hypertension, smoking, alcohol consumption, gestational age, birth weight and BMI child.

Ħ Only analyzed in women already parenting (N = 1,302).

a) Each 1-unit increase in depressive symptoms/state anxiety/pregnancy-related anxiety/parenting daily hassles increases the mean systolic BP/diastolic BP/MAP with β mmHg.

b) The beta’s per job strain-category indicate the mean difference in mmHg as compared to the low job strain-category.

c) The beta’s per cumulative stress score-category indicate the mean difference in mmHg as compared to the no stress-category.

Like depressive symptoms, state anxiety was associated with higher systolic BP, diastolic BP and MAP in the minimally adjusted regression models (Table 2), and it significantly increased the odds of the child being hypertensive (all p<0.01) (Table 3). Again, the associations were attenuated and lost significance upon full adjustment for confounders.

**Table 3:** Associations (odds ratios) between maternal prenatal stressors and Hypertension in the child at age five-seven (N = 2968).

	Model 1		Model 2	
	OR	95% CI	OR	95% CI
	Hypertension‡			
Depressive symptoms^a^	1.02	(1.00;1.03)	1.00	(0.98;1.02)
State anxiety^a^	1.02**	(1.01;1.04)	1.01	(1.00;1.03)
Pregnancy-related anxiety^a^	1.05**	(1.02;1.09)	1.02	(0.99;1.06)
Parenting daily hassles Ħ^a^	1.01	(0.98;1.05)	1.00	(0.97;1.03)
*Job strain^b^*				
Low job strain (reference)	0	0	0	0
No job	1.2	(0.7;1.9)	0.8	(0.5;1.3)
Moderate job strain	1.1	(0.7;1.7)	1.1	(0.7;1.7)
High job strain	1.0	(0.6;1.7)	0.9	(0.5;1.6)
*Cumulative stress score^c^*				
No stress (reference)	0	0	0	0
1 Stressor	1.5**	(1.0;2.0)	1.4	(1.0;2.0)
2 Stressors	0.8	(0.5;1.4)	0.7	(0.4;1.1)
3–4 Stressors	2.3**	(1.2;4.2)	1.7	(0.9;3.4)

** p<0.05.

* To model 1, sex, height and age of the child at measurement were added as covariates.

† To model 2, additionally added covariates are: maternal and paternal (family) hypertension, maternal age, ethnicity, pre-pregnancy BMI, educational level, primiparity, maternal hypertension, smoking, alcohol consumption, gestational age, birth weight and BMI child.

‡ The definition of hypertension was sex, height and age-specific: therefore, these covariates were not added.

Ħ Only analyzed in women already parenting (N = 1,302).

Pregnancy related anxiety was associated with higher systolic BP (p<0.01), MAP (p = 0.02) and it significantly increased the odds of the child being hypertensive (p<0.01). The associations did not remain significant after full adjustment for confounders.

Parenting daily hassles were not associated with any of the outcomes measures, both in the minimally and the fully adjusted models.

Children of mothers in the no job-category had a significantly higher mean diastolic BP and MAP (p<0.01 and p = 0.02 respectively) as compared to women who did have jobs, and experienced low job strain, but this association did not remain significant after full adjustment for confounders.

The sex of the child was not found to be an effect modifier in the associations between the individual stress scales and the blood pressure measures (all p≥0.13).

### Cumulative Stress Score

In minimally adjusted analyses, mean systolic BP, diastolic BP and MAP were significantly higher in children from mothers in the highest cumulative stress category (3–4 stressors) as compared to mothers in the no stress category (p<0.01). The associations of the highest stress category with systolic BP, diastolic BP and MAP remained after full adjustment for confounders: Children from mothers in the highest stress category, which denotes the presence of three or four stressors, had a borderline significant 1.5 mmHg higher systolic BP (p = 0.046), a 1.5 mmHg higher diastolic BP (p = 0.04) and a 1.5 higher MAP (p = 0.02), compared with the children of mothers in the no stress category. However, Bonferroni correction for multiple testing rendered all these associations between the cumulative stress score and offspring blood pressure non-significant (α 0.05/6 comparisons, means p<0.008 equals statistical significance on these specific ANOVAs). The odds ratios (OR) for hypertension was significantly increased in the group with one stressor as well as in the group with 3–4 stressors (p = 0.02), but these associations did not remain statistically significant after full adjustment for confounders.

The sex of the child was not found to be an effect modifier in the associations between the cumulative stress score and the blood pressure measures. The interaction term yielded the following p-values: 0.52 for systolic BP; 0.69 for diastolic BP and 0.78 for MAP. This indicated that the association between maternal stress and BP was similar between boys and girls and therefore, the analyses were not repeated with stratification by sex.

## Discussion

This study shows, for the first time, that the prenatal presence of multiple psychosocial stressors was associated with higher systolic and diastolic BP and MAP in the child at the age of 5–7 years. Besides increased blood pressure within the healthy ranges, the risk for hypertension was also increased by maternal prenatal exposure to multiple psychological stressors. However, this increase did not remain statistically significant after adjustment for confounders.

### Comparison to Existing Literature

Our findings are in line with previous studies. In rats, the offspring of prenatally stressed mothers show a higher BP in adulthood [37]. Also, higher maternal cortisol early in pregnancy appears to be associated with both increased vascular resistance and lower arterial elasticity in human children aged 5–7 [51], [52]. Similar to our results, after adjustment for confounders, these studies did not find an association with hypertension. Possibly, the prevalence of hypertension in these young children is still relatively low, resulting in insufficient power to produce statistically significant findings.

In contrast with our study, Holst et al. [37] showed increased diastolic blood pressure only in male offspring of prenatally stressed rats, where we show increased diastolic blood pressure in both sexes. And potentially in contrast with Holst et al., McCormick et al. observed that the effect of prenatal stress on HPA function is considerably stronger in female rat offspring [10]. The results regarding sex-specific programming in rats may, however, not be fully comparable to ours, because it has been argued that differences in response due to sex may differ between species [53].

Adams et al. reiterated the association between pediatric hypertension and learning and attention problems [54]: the causal direction however remains undetermined. We therefore briefly explored internalizing and externalizing behavioral problems within the maternal stress-offspring BP association. Behavioral problems slightly attenuated the association between high prenatal cumulative stress and offspring systolic BP, but because it is not yet clear whether they are mediating, or whether they are situated further down the causal chain, we did not add them as confounders to the models in this study.

### Strengths & Limitations

Our findings do not reflect a linear increase in BP with an increasing number of stressors: merely being a mother in the highest stress category was associated with the offspring’s BP. As it takes substantial, chronic stress to influence the fetal HPA axis [6], [7], [16], there may be a threshold responsible for this seemingly non-linear association. In the minimally adjusted model, we also observed an increased risk of hypertension in the *lowest* stress category, which was not in line with the rest of our findings. This group did not show a deviant demographic constitution to explain this result. This reminds us that there is always a chance of residual confounding being present, despite the adjustment for many potential confounders. One source of residual confounding may be postnatal stress, a measure we were not able to adjust for in the current study. Also, the levels of significance need to be interpreted with caution, because multiple comparisons are made and Bonferroni correction for multiple testing rendered all the associations non-significant. Confirmation of the associations reported is thus needed.

The large sample size is a strength of the current study, but as in most cohort studies, selective loss to follow-up was present. In the ABCD cohort as a whole, stress was more prevalent than it is in the current subgroup, which is now a slightly healthier reflection of the population (i.e. higher educational level, lower BMI and lower stress levels). This could indicate that, had the follow-up been 100%, the associations observed in the present study may have been more pronounced. In addition, this means that the proportion of pregnant women in the highest stress category might be larger at population level.

The method of assessment of maternal stress varies greatly between studies in the DOHAD (Developmental Origins of Health and Disease) field [55]. We had common psychosocial stress constructs available from our cohort, assessed with the appropriate validated instruments. In previous research we applied latent class (cluster) analysis, and were able to define different clusters of women with typical stress patterns (Loomans & van Dijk et al. 2012). Psychosocial stress during pregnancy is related to adverse birth outcomes: results from a large multi-ethnic community-based birth cohort. Accepted at European Journal of Public Health). This method did however not enable us to pinpoint the group of women experiencing different, multiple forms of high stress, which is what our currently applied cumulative score does allow.

Both depression and anxiety were viable options for inclusion in the cumulative stress score. We did however choose depression, because we already included pregnancy-related anxieties. Using the anxiety scale instead of the depression scale hardly changed the percentages of subjects in the different categories (e.g. 3–4 stressors: 4.2 to 4.1%). 5% of the respondents moved up one category, and 4% moved down a category. The correlation between the two different cumulative scores was 0.94 (p<0.001). The associations and levels of statistical significance between the highest stress category and blood pressure measures were comparable between the two definitions of the cumulative score.

The automatic oscillometric assessment of BP may have eliminated potential inter-observer differences, although it should be noted that the reference values for defining hypertension have been obtained by the auscultatory method. The oscillometric method tends to report higher values [56]. Also, the American reference values are in general several mmHg lower than those measured in smaller, European samples [57], [58]. Despite the resulting potential overestimation of hypertension, we did not observe statistically significant associations with hypertension.

### Potential Mechanisms

Several mechanisms may explain the association between prenatal maternal stress and offspring blood pressure. Evidence has shown that prenatal chronic stress influences the maternal HPA axis, leading to increased levels of cortisol, which may influence the development of the fetal HPA axis [6], [7], [16]. Alternatively, or additionally, maternal anxiety may increase the permeability of the placenta to cortisol due to downregulation of placental 11-Beta hydroxysteroid dehydrogenase activity [16], [22], [23]. A study in non-human primates directly linked prenatal dexamethasone exposure to increased blood pressure, and an exaggerated cortisol response (HPA-response) to mild stress, in the offspring [59]. In human and animal studies, prenatal exposure to glucocorticoid has been associated with BP at birth, fetal and adult vascular responses to vasoconstrictors (microvascular dysfunction), and has also resulted in alterations of some aspects of the local renin-angiotensin system (particularly in the kidney and brain) [3], [4], [11]–[13], [27], [60]. Similar results have also been reported after administering subtle cortisol disturbances, which were comparable to disturbances as a result of mild stress. In rats, this resulted in a nephron deficit and development of hypertension in the offspring [61]. Those results support the idea that prenatal cortisol is predictive of offspring blood pressure through adaptations in the renin-angiotensin system.

Seemingly contradicting a pathway through increased maternal cortisol is the lack of correlation between self-reported and physiological stress, which is not uncommon in literature [62], [63]. Diego et al. did report correlations between maternal cortisol and psychological stress measures, but the highest correlation coefficient was 0.4 (cortisol and depressive symptoms) [64]. Differences between studies may however be attributed to methodological differences. In most studies, even though assessment took place at several time points throughout pregnancy, single cortisol measures were taken: In a recent study by Entringer et al. the use of a repeated ambulatory assessments of cortisol led to the detection of an association with maternal negative affect [65]. Therefore, increased maternal cortisol still offers a viable potential pathway by which maternal stress may cause fetal programming.

An alternative mechanism involves inflammation markers: maternal psychosocial factors can contribute to higher circulating levels of inflammation markers, like C-reactive protein (CRP) and the proinflammatory cytokines IL-1b, IL-6, and TNF-a, and lower circulating levels of the antiinflammatory cytokine IL-10 and ex vivo endotoxin (lipopolysaccharide)-stimulated levels of IL-1b and IL-6 [19], [66]–[68]. The influence of maternal inflammation-linked preterm birth on the development of adult cardiovascular disease has not been extensively investigated [20]. Direct programming effects of maternal inflammation, thus not mediated by preterm birth, have also been suggested: In a review by Entringer et al. [69] offspring body composition, metabolic function and obesity risk are linked to prenatal stress biology, with an important role for immunological pathways. Unfortunately, we do not have data from repeated (ambulatory) assessments of cortisol in the pregnant women, or data from inflammatory biomarkers. We can therefore not elucidate which of these potential mechanisms may play a role in the in this paper reported associations between prenatal stress and offspring blood pressure.

Intra-uterine growth retardation could be another mechanism through which maternal stress affects offspring BP. The association between maternal stress and fetal growth restriction has been well documented [55], [70], [71], as well as the association between low birth weight and an increased risk of high BP in later life [72]–[75]. In our study, prenatal stress was, however, not associated with lower birth weight (data not shown). Therefore, our results do not indicate a strong mediating role of size at birth in the association between prenatal stress and offspring BP. But, confirming existing literature, lower birth weight was significantly associated with higher offspring systolic and diastolic BP, MAP and hypertension in our study sample, independently from prenatal stress and relevant confounders (data not shown).

Furthermore, measuring BP could be experienced as more stressful in children from mothers with high-stress scores: an argument that is supported by evidence showing that prenatal stress does not have an effect on baseline stress (cortisol) parameters, because the sensitivity of the HPA axis has been affected. There would only be an effect on stress reactivity, causing hyper reactivity during stress tests. For example, increased cardiovascular reactivity to stress was observed in babies of smokers and babies born preterm [76] and increased cardiac sympathetic activation was observed in babies who were small at birth [77], [78].

Alternatively, maternal (pre-existing) hypertension may be a factor explaining the association between prenatal stress and the offspring’s BP, because literature, as well as the significantly different prevalence of hypertension between the cumulative stress categories in our study (data not shown), suggest that stress is associated with higher BP [79]. Maternal hypertension was incorporated in our fully adjusted model and removing this variable did not change the observed association between prenatal stress and offspring BP, indicating that maternal hypertension does not play a mediating role. BMI of the child at age five-seven did slightly mediate the associations between prenatal stress and offspring blood pressure: When BMI was omitted from the fully adjusted regression models, the beta’s of the highest stress category changed from 1.5 (p = 0.046) to 1.7 (p = 0.03) for systolic BP, from 1.5 (p = 0.04) to 1.6 (p = 0.03) for diastolic BP and from 1.5 (p = 0.02) to 1.6 for MAP (p = 0.02). The association with hypertension remained non-significant.

### Timing of Stress: Window of Sensitivity

The timing of stress assessment in pregnancy may be key to finding potential fetal programming effects. In the rat, exposure to glucocorticoids in the final week of gestation causes adult hypertension in the offspring [9], whereas the window of sensitivity in sheep is earlier in gestation [14]. The assessment of stress in the current study took place in the first week of the second trimester, thus assessing experienced stress in the preceding first trimester-weeks. The first trimester is often considered the trimester with the highest fetal vulnerability because of the development of critical, basal systems, including the formation of nephrons [77], [80]. Unfortunately, we did not have multiple measurements throughout pregnancy to test this hypothesis.

As some studies have illustrated, as pregnancy advances, a woman’s psychological response to stress is blunted, which may be due to a decreasing capacity for the physiologic (neuroendocrine) stress response [8]. Despite the mean decrease, anxiety and depression are stable across the period of pregnancy [81]. Therefore, our observed associations with first trimester stress might not necessarily be bound to the first trimester. In sum, the stage of pregnancy may play a role in the association between maternal stress and fetal programming [71], although the exact windows of sensitivity are yet to be identified, especially in humans.

### Implications

Our findings may suggest that the presence of multiple psychosocial stressors during pregnancy is associated with higher systolic and diastolic BP and MAP in the child, as early as the age of five years. As childhood BP is likely to track into adulthood [82], [83], we can expect the currently reported increased blood pressure to persist and possibly become more pronounced in later life: the impact on hypertension and resulting CVD risk could be compelling. Results from the Bogalusa Heart Study showed that twice the expected number of subjects whose blood pressure levels were in the highest quintile at childhood, remained in the highest quintile 15 years later [83]. In our study, the observed effect size was a 1.5 mmHg increase on both systolic and diastolic BP in children of mothers classified as high-stress. This might not seem much, but if all children experienced a 1.5 mmHg increase, then 49% (systolic BP) and 48.5% (diastolic BP) would be in the highest quintile. In comparison, in the Bogalusa Heart Study, this was 40% (systolic BP) and 37% (diastolic BP).

Prenatal exposure to stress could be indicated as a factor underlying the pathogenesis of cardiovascular disease, but the reported association with offspring blood pressure requires further investigation. Prevention of maternal stress in the child’s early stages of life may be valuable to improve cardiovascular health later in life.
